# Survival without major morbidity in very preterm infants (<32 weeks’ gestation): prevalence, temporal trends, and associated factors in a single-center retrospective cohort

**DOI:** 10.3389/fped.2026.1871207

**Published:** 2026-08-28

**Authors:** Liu Yang, Yikun Zhang, Chuan Nie, Zhiren Chen, Yumei Jian, Di Zhong, Yuanhan Qin

**Affiliations:** 1 The First Affiliated Hospital of Guangxi Medical University/Difficult and Critical Illness Center, Pediatric Clinical Medical Research Center of Guangxi, Nanning, Guangxi Zhuang Autonomous Region, China; 2 Department of Neonatology, Guangdong Women and Children Hospital, Guangzhou, Guangdong, China; 3 National Key Clinical Specialty Construction Project/Guangdong Neonatal ICU Medical Quality Control Center, Guangzhou, Guangdong, China; 4 Wushan Subdistrict Community Health Service Center, Guangzhou, Guangdong, China; 5 Department of Pediatrics, Guangdong Women and Children Hospital, Guangzhou, Guangdong, China

**Keywords:** associated factors, discharge against medical advice, survival without major morbidity, temporal trends, very preterm infant

## Abstract

**Objective:**

To determine the proportion of very preterm infants (VPIs) surviving without major morbidity (SWMM), assess temporal trends from 2021 to 2025, and identify associated factors.

**Methods:**

This single-center retrospective cohort study included all inborn VPIs admitted to the neonatal intensive care unit (NICU) of Guangdong Women and Children Hospital between January 2021 and October 2025. Primary analyses were performed among infants receiving complete care, whereas infants discharged against medical advice (DAMA) were analyzed separately. SWMM was defined as survival without bronchopulmonary dysplasia (BPD), severe brain injury, necrotizing enterocolitis (NEC) stage II or higher, late-onset sepsis (LOS), or severe retinopathy of prematurity (ROP). Temporal trends were evaluated using logistic regression with gestational age (GA)-adjusted annual rates estimated by marginal standardization. Factors associated with SWMM were assessed using multivariable logistic regression.

**Results:**

A total of 1,172 VPIs were included, with a median GA of 30.0 weeks (IQR, 28.6–31.1) and a median birth weight of 1,270 g (IQR, 1,050–1,500). In the complete-care cohort (*n* = 1,094), survival was 95.5% (1,045/1,094) and SWMM was 59.9% (655/1,094). From 2021 to 2025, survival remained high and stable. After GA adjustment, BPD, severe ROP, and LOS increased over time, whereas SWMM declined. Multivariable analysis showed that higher GA (per week: OR 2.62, 95% CI 2.30–3.01) and greater birth weight z score (per SD: OR 2.13, 95% CI 1.76–2.59) were associated with higher odds of SWMM, whereas male sex (OR 0.66, 95% CI 0.48–0.91), additional surfactant doses (per dose: OR 0.47, 95% CI 0.37–0.59), and a 5-minute Apgar score ≤7 (OR 0.27, 95% CI 0.10–0.69) were associated with lower odds. DAMA occurred in 6.7% of VPIs (78/1,172) and decreased with increasing GA, from 30.0% at ≤25 weeks to 1.9% at 31 weeks. The estimated post-DAMA survival rate was 24.4% (19/78).

**Conclusion:**

Survival among VPIs remained high and stable, whereas SWMM remained suboptimal and declined over time. The increasing burden of BPD and severe ROP underscores the importance of improving the quality of survival. Strategies including GA-stratified care, optimized perinatal stabilization, early respiratory management, and reduced DAMA may improve outcomes in this high-risk population.

## Introduction

Preterm birth remains a leading cause of neonatal mortality and long-term neurodevelopmental impairment worldwide, particularly among very preterm infants (VPIs, <32 weeks’ gestation). With advances in perinatal medicine and neonatal intensive care, survival rates of VPIs have improved substantially over recent decades (1–10). Previous studies have shown that major morbidities of VPIs, including bronchopulmonary dysplasia (BPD), necrotizing enterocolitis (NEC), and retinopathy of prematurity (ROP), are closely associated with adverse long-term outcomes. Accordingly, survival without major morbidity (SWMM) has emerged as a crucial composite endpoint (8, 11–14).

Substantial heterogeneity in SWMM has been reported across studies, with rates varying widely from 40% to 54% (8, 9, 11, 12, 15, 16). This variability is largely attributable to differences in gestational age (GA) distribution, SWMM definitions, case mix, clinical practice, and the management of infants discharged against medical advice (DAMA) (17). Consequently, estimates from other cohorts are not directly comparable, underscoring the need for continuous, standardized evaluation within individual healthcare systems to monitor temporal trends and identify opportunities for quality improvement. As a provincial referral center in South China, Guangdong Women and Children Hospital (GDWCH) manages approximately 18,000 deliveries annually and provides tertiary neonatal care for a large number of VPIs, including complex maternal or fetal conditions. However, SWMM has not previously been systematically evaluated. Although survival among VPIs appeared to remain high at our center, clinicians observed an increase in BPD and severe ROP in recent years. This divergence between survival and morbidity raised the question of whether high survival translated into favorable morbidity-free survival, highlighting the need for a systematic evaluation of SWMM.

This study aimed to determine the proportion of SWMM among VPIs at GDWCH, assess its temporal trends from 2021 to 2025, and identify factors associated with SWMM, with the goal of optimizing clinical care, improving neonatal outcomes, and supporting prognostic counseling for families of vulnerable preterm infants.

## Methods

### Study design and setting

This was a single-center retrospective cohort study conducted in the neonatal intensive care unit (NICU) of GDWCH. The study period spanned from January 2021 to October 2025.

### Study population

All infants with a GA <32 weeks admitted to the NICU during the study period were screened (overall NICU cohort, *n* = 1,172). Primary analyses were restricted to infants who received complete medical care until physician-recommended discharge or in-hospital death (*n* = 1,094). Infants subject to DAMA were excluded from the primary analyses but analyzed as a separate subgroup (*n* = 78). Infants who underwent obstetric termination of pregnancy or failed delivery-room resuscitation and were not admitted to the NICU were excluded.

### Data sources and variables

Clinical data were extracted from the electronic medical record system and the NICU database using a standardized data abstraction protocol. Extracted variables included maternal and perinatal characteristics, delivery-room interventions, and in-hospital NICU clinical data.

### Outcomes and definitions

The primary outcome was SWMM, defined as survival to discharge without any of the major morbidities including BPD, severe brain injury, NEC ≥ stage II, LOS, and severe ROP (13). BPD was defined as requirement for oxygen supplementation or respiratory support at 36 weeks’ postmenstrual age among infants surviving to that time point (18). Severe brain injury was defined as intraventricular hemorrhage (IVH) grade III-IV and/or cystic periventricular leukomalacia (PVL) based on neuroimaging findings (19). NEC ≥ stage II was defined according to the modified Bell's staging criteria (20). LOS was defined as culture-proven sepsis occurring after 72 h of life (21). Severe ROP was defined as ROP ≥ stage 3 or receiving treatment, including intravitreal injection or laser photocoagulation (22). Secondary outcomes included survival, major morbidity burden, and survival with one or ≥2 major morbidities.

DAMA was defined as parent-initiated withdrawal of care prior to physician-recommended discharge. Infants discharged in a moribund condition for palliative purposes were classified as in-hospital deaths (23). Because systematic post-discharge follow-up data were unavailable for infants with DAMA, survival after DAMA was estimated using prespecified criteria rather than directly observed. Specifically, infants requiring invasive or noninvasive ventilation, inotropic support, or exclusive parenteral nutrition at discharge were presumed non-survivors (24). This approach was used only for descriptive estimation and was not included in the primary complete-care analysis.

### Statistical analysis

Continuous variables were summarized as medians with interquartile ranges (IQRs), and categorical variables as frequencies and percentages. To estimate GA-adjusted annual rates, birth year was modeled as a categorical variable and GA was included as a continuous covariate. Adjusted annual probabilities and corresponding 95% CIs were estimated as average marginal predicted probabilities, standardized to the observed GA distribution of the complete-care cohort. To formally assess linear temporal trends, separate logistic regression models were fitted with calendar year treated as a continuous numeric predictor. Crude models included calendar year only, whereas adjusted models additionally included GA. Results are presented as OR per one-year increase, with corresponding 95% CI and *P* values for trend.

Factors associated with SWMM were evaluated using multivariable logistic regression model in the complete-care cohort. Candidate variables were selected *a priori* based on clinical relevance, biological plausibility, evidence from previous studies, and their availability before birth or during the early postnatal period. BW was modeled as a sex- and GA-specific z score derived from the INTERGROWTH-21st newborn size standards to minimize collinearity (25). Model diagnostics and performance were evaluated comprehensively. Multicollinearity was assessed using variance inflation factors (VIFs), with VIF values <5 indicating no evidence of substantial multicollinearity. Model discrimination was quantified by the area under the receiver operating characteristic curve (AUC), overall prediction accuracy by the Brier score, and goodness-of-fit by the Hosmer-Lemeshow test. Sensitivity analyses were performed by refitting the primary model after excluding either GA or BW z score to assess the robustness of the estimated associations.

All statistical analyses were conducted using R software (version 4.5.2). A two-sided *P* value <0.05 was considered statistically significant.

### Ethics statement

The study protocol was approved by the Institutional Ethics Committee of GDWCH (approval number: 20261021). The requirement for informed consent was waived due to the retrospective design and use of de-identified routinely collected clinical data.

## Results

### Study cohort

From January 2021 to October 2025, a total of 1,172 VPIs were included. Of these, 78 infants (6.7%) were DAMA, while 1,094 (93.3%) completed their prescribed medical care and comprised the primary analytic cohort.

### Baseline characteristics and perinatal/NICU practices

Among all admitted 1,172 VPIs, the median GA was 30.0 weeks (IQR, 28.6–31.1), and the median BW was 1,270 g (IQR, 1,050–1,500). Overall, 51.5% were born at 30–31 weeks’ gestation, only 3.4% were born at ≤25 weeks.

BW increased progressively across GA strata, rising from a median of 700.0 g at ≤25 weeks to 1,530.0 g at 31 weeks. Lower GA was associated with greater need for delivery room resuscitation and more intensive postnatal care. Delivery room intubation rates decreased from 67.5% at ≤25 weeks to 10.5% at 31 weeks. Similarly, the median duration of invasive ventilation declined from 18.0 to 0.0 days, and the median NICU length of stay decreased from 101.0 to 38.0 days across GA strata (Table 1).

**Table 1:** Baseline characteristics, perinatal interventions, and early NICU data among all NICU-admitted VPIs, stratified by GA.

Variable	Total (*n* = 1,172)	GA ≤25 (*n* = 40)	GA 26 (*n* = 48)	GA 27 (*n* = 98)	GA 28 (*n* = 180)	GA 29 (*n* = 202)	GA 30 (*n* = 234)	GA 31 (*n* = 370)
Infant and maternal baseline characteristics								
Male, *n* (%)	670 (57.2)	20 (50.0)	27 (56.2)	52 (53.1)	106 (58.9)	111 (55.0)	145 (62.0)	209 (56.5)
SGA, *n* (%)	74 (6.3)	6 (15.0)	2 (4.2)	1 (1.0)	7 (3.9)	6 (3.0)	15 (6.4)	37 (10.0)
Multiple birth, *n* (%)	444 (37.9)	17 (42.5)	24 (50.0)	41 (41.8)	48 (26.7)	56 (27.7)	99 (42.3)	159 (43.0)
Primigravida, *n* (%)	367 (31.3)	7 (17.5)	12 (25.0)	23 (23.5)	45 (25.0)	69 (34.2)	82 (35.0)	129 (34.9)
PIH, *n* (%)	193 (16.5)	1 (2.5)	3 (6.2)	12 (12.2)	24 (13.3)	36 (17.8)	40 (17.1)	77 (20.8)
GDM, *n* (%)	327 (27.9)	4 (10.0)	10 (20.8)	26 (26.5)	56 (31.1)	68 (33.7)	67 (28.6)	96 (25.9)
IVF, *n* (%)	241 (20.6)	4 (10.0)	22 (45.8)	30 (30.6)	42 (23.3)	29 (14.4)	49 (20.9)	65 (17.6)
Maternal age, y, M (Q1, Q3)	31.0 (28.0, 35.0)	30.0 (28.0, 33.0)	30.50 (29.0, 37.0)	32.0 (29.0, 36.0)	32.0 (28.8, 35.0)	31.0 (28.0, 35.0)	31.0 (28.0, 35.0)	31.0 (28.0, 34.0)
Birth length, cm, M (Q1, Q3)	37.0 (34.9, 39.0)	30.0 (28.0, 32.0)	32.0 (31.0, 34.3)	34.0 (32.0, 35.0)	36.0 (34.0, 37.0)	37.0 (35.0, 38.0)	38.0 (36.0, 40.0)	39.0 (37.0, 41.0)
Birth head circumference, cm, M (Q1, Q3)	27.0 (25.0, 28.0)	23.0 (21.0, 24.0)	24.0 (22.0, 24.3)	25.0 (24.0, 26.0)	26.0 (25.0, 27.0)	27.0 (26.0, 28.0)	28.0 (26.5, 29.0)	28.0 (27.0, 29.0)
BW, g, M (Q1, Q3)	1,270.0 (1,050.0, 1,500.0)	700.0 (600.0, 810.0)	855.0 (800.0, 947.5)	1,015.0 (900.0, 1,097.5)	1,115.0 (1,000.0, 1,222.5)	1,225.0 (1,092.5, 1,350.0)	1,400.0 (1,250.0, 1,550.0)	1,530.0 (1,322.5, 1,717.5)
Perinatal interventions and findings								
Cesarean delivery, *n* (%)	751 (64.1)	11 (27.5)	17 (35.4)	45 (45.9)	111 (61.7)	139 (68.8)	168 (71.8)	260 (70.3)
ANS, *n* (%)	961 (82.0)	23 (57.5)	34 (70.8)	68 (69.4)	151 (83.9)	174 (86.1)	199 (85.0)	312 (84.3)
Grade III MSAF, *n* (%)	31 (2.6)	1 (2.5)	5 (10.4)	6 (6.1)	7 (3.9)	4 (2.0)	3 (1.3)	5 (1.4)
PROM > 18 h, *n* (%)	294 (25.1)	6 (15.0)	15 (31.2)	25 (25.5)	45 (25.0)	62 (30.7)	55 (23.5)	86 (23.2)
DCC, *n* (%)	250 (21.3)	6 (15.0)	7 (14.6)	22 (22.4)	35 (19.4)	45 (22.3)	50 (21.5)	85 (23.0)
PPV, *n* (%)	1,106 (94.4)	36 (90.0)	47 (97.9)	92 (93.9)	170 (94.4)	194 (96.0)	220 (94.4)	347 (93.8)
Intubation, *n* (%)	222 (19.0)	27 (67.5)	21 (43.8)	27 (27.6)	41 (22.8)	31 (15.3)	36 (15.5)	39 (10.5)
Chest compression, *n* (%)	37 (3.2)	8 (20.0)	1 (2.1)	5 (5.1)	7 (3.9)	8 (4.0)	4 (1.7)	4 (1.1)
Epinephrine, *n* (%)	28 (2.4)	7 (17.5)	1 (2.1)	2 (2.0)	6 (3.3)	6 (3.0)	1 (0.4)	5 (1.4)
First min Apgar score ≤7, *n* (%)	248 (21.2)	27 (67.5)	24 (50.0)	31 (31.6)	52 (28.9)	37 (18.3)	28 (12.0)	49 (13.2)
Five-min Apgar score ≤7, *n* (%)	58 (4.9)	13 (32.5)	6 (12.5)	4 (4.1)	12 (6.7)	6 (3.0)	5 (2.1)	12 (3.2)
Postnatal interventions and findings after admission to NICU								
Admission hypothermia, *n* (%)	286 (24.4)	17 (42.5)	17 (35.4)	22 (22.4)	57 (31.7)	48 (23.8)	39 (16.7)	86 (23.2)
Postnatal corticosteroid use^a^, *n* (%)	65 (5.9)	7 (28.0)	5 (13.9)	17 (19.1)	13 (8.0)	5 (2.5)	9 (4.0)	9 (2.5)
Caffeine^b^, *n* (%)	1,043 (97.0)	15 (68.2)	35 (97.2)	84 (97.7)	155 (97.5)	196 (100.0)	214 (97.3)	344 (96.6)
Insulin^b^, *n* (%)	50 (4.7)	5 (22.7)	7 (19.4)	11 (12.8)	10 (6.3)	9 (4.6)	1 (0.5)	7 (2.0)
Ibuprofen^b^, *n* (%)	153 (14.2)	10 (45.5)	18 (50.0)	30 (34.9)	44 (27.7)	20 (10.2)	15 (6.8)	16 (4.5)
Vasoactive agent^b^, *n* (%)	292 (27.2)	19 (86.4)	21 (58.3)	41 (47.7)	54 (34.0)	53 (27.0)	40 (18.2)	64 (18.0)
Concomitant use of ≥2 intravenous antibiotics^b^, *n* (%)	696 (64.7)	21 (95.5)	36 (100.0)	72 (83.7)	120 (75.5)	137 (69.9)	140 (63.6)	170 (47.8)
Breast milk feeding^b^, *n* (%)	536 (49.9)	12 (54.5)	19 (52.8)	43 (50.0)	98 (61.6)	101 (51.5)	99 (45.0)	164 (46.1)
Invasive ventilation^c^, d, M (Q1, Q3)	0.0 (0.0, 6.0)	18.0 (5.0, 33.3)	6.0 (0.0, 11.5)	7.0 (1.0, 12.5)	3.0 (0.0, 7.0)	0.0 (0.0, 7.0)	0.0 (0.0, 3.0)	0.0 (0.0, 2.0)
Noninvasive ventilation^c^, d, M (Q1, Q3)	14.0 (6.0, 28.0)	42.5 (0.0, 56.3)	35.5 (11.5, 49.8)	37.0 (26.0, 48.0)	27.0 (20.0, 37.0)	18.0 (11.0, 27.0)	10.0 (5.0, 17.8)	7.0 (4.0, 13.0)
Duration of first course of invasive ventilation^c^, d, M (Q1, Q3)	0.0 (0.0, 5.0)	13.0 (5.0, 30.8)	4.5 (0.0, 7.0)	6.5 (1.0, 11.0)	2.0 (0.0, 5.8)	0.0 (0.0, 6.0)	0.0 (0.0, 3.0)	0.0 (0.0, 2.0)
PS doses^a^, M (Q1, Q3)	1.0 (0.0, 1.0)	2.0 (1.0, 2.0)	1.0 (1.0, 1.0)	1.0 (1.0, 2.0)	1.0 (1.0, 1.0)	1.0 (0.0, 1.0)	1.0 (0.0, 1.0)	0.0 (0.0, 1.0)
NICU stay^c^, d, M (Q1, Q3)	51.0 (38.0, 65.0)	101.0 (33.8, 117.0)	82.0 (67.0, 91.0)	74.0 (67.8, 83.0)	64.0 (56.0, 74.0)	55.0 (49.8, 63.0)	46.0 (41.0, 53.8)	38.0 (32.0, 47.0)
Number of RBC transfusions^b^, M (Q1, Q3)	0.0 (0.0, 1.0)	1.5 (1.0, 2.0)	1.0 (0.0, 2.0)	1.0 (0.0, 1.0)	0.0 (0.0, 1.0)	0.0 (0.0, 1.0)	0.0 (0.0, 0.0)	0.0 (0.0, 0.0)

NICU, neonatal intensive care unit; SGA, small for gestational age; PIH, pregnancy-induced hypertension; GDM, gestational diabetes mellitus; IVF, *in vitro* fertilization; ANS, antenatal steroids; MSAF, meconium-stained amniotic fluid; PROM, prolonged rupture of membranes; DCC, deferred umbilical cord clamping; PPV, positive pressure ventilation; GA, gestational age; BW, birth weight; VPI, very preterm infant.

a Calculated among infants with a hospital length of stay >7 days (*n* = 1,096).

b Calculated among infants with a hospital length of stay >14 days (*n* = 1,075).

c Calculated among infants receiving complete care (*n* = 1,094).

### Survival, major morbidity, and SWMM

**Figure 1:**
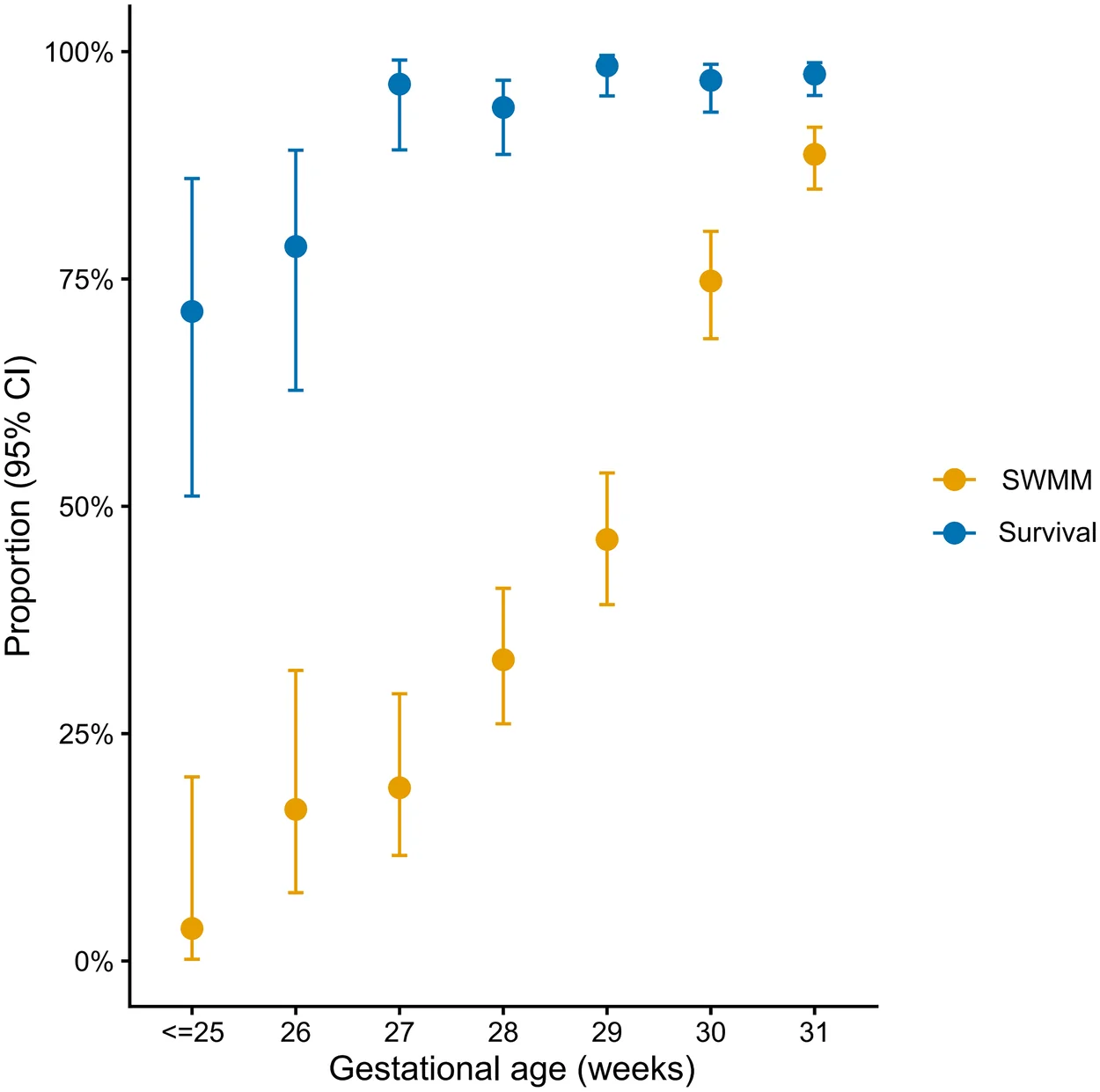
Survival and survival without major morbidity (SWMM) among very preterm infants receiving complete care.

With increasing GA, survival increased from 71.4% at ≤25 weeks to 97.5% at 31 weeks, while SWMM increased from 3.6% to 89.3%. Importantly, the gap between survival and SWMM widened sharply with decreasing GA, particularly among infants born at ≤29 weeks, highlighting the substantial burden of major morbidity among those infants (Figure 1).

The burden of major morbidities varied substantially across GA strata. Overall, BPD was the most common major morbidity, occurring in 303 infants (27.7%), and was most frequent among infants born at 27 weeks (69.0%), but decreased to 4.7% at 31 weeks. Severe ROP also declined with increasing GA, from 50.0% at ≤25 weeks to 3.3% at 31 weeks. Survival with ≥2 major morbidities showed the most pronounced GA-related gradient, decreasing from 64.3% at ≤25 weeks to 2.2% at 31 weeks. In contrast, survival with 1 major morbidity peaked among infants born at 27–28 weeks (41.7% and 47.2%, respectively) (Table 2).

**Table 2:** Major morbidities among VPIs receiving complete care, stratified by GA.

Variable	Total (*n* = 1,094)	GA ≤25 (*n* = 28)	GA 26 (*n* = 42)	GA 27 (*n* = 84)	GA 28 (*n* = 163)	GA 29 (*n* = 192)	GA 30 (*n* = 222)	GA 31 (*n* = 363)
BPD, *n* (%)	303 (27.7)	18 (64.3)	21 (50.0)	58 (69.0)	86 (52.8)	71 (37.0)	32 (14.4)	17 (4.7)
LOS, *n* (%)	60 (5.5)	10 (35.7)	1 (2.4)	9 (10.7)	10 (6.1)	12 (6.2)	9 (4.1)	9 (2.5)
NEC ≥ stage 2, *n* (%)	65 (5.9)	5 (17.9)	3 (7.1)	10 (11.9)	11 (6.7)	17 (8.9)	13 (5.9)	6 (1.7)
Severe brain injury, *n* (%)	76 (6.9)	10 (35.7)	12 (28.6)	12 (14.3)	12 (7.4)	19 (9.9)	9 (4.1)	2 (0.6)
Severe ROP, *n* (%)	89 (8.1)	14 (50.0)	14 (33.3)	16 (19.0)	10 (6.1)	13 (6.8)	10 (4.5)	12 (3.3)
Survival with 1 major morbidity, *n* (%)	255 (23.3)	1 (3.6)	9 (21.4)	35 (41.7)	77 (47.2)	75 (39.1)	34 (15.3)	24 (6.6)
Survival with ≥2 major morbidities, *n* (%)	135 (12.3)	18 (64.3)	17 (40.5)	30 (35.7)	22 (13.5)	25 (13.0)	15 (6.8)	8 (2.2)

NEC, necrotizing enterocolitis; ROP, retinopathy of prematurity; BPD, bronchopulmonary dysplasia; LOS, late-onset sepsis; GA, gestational age; VPI, very preterm infant.

### Crude and GA-adjusted temporal trends in survival, major morbidity, and SWMM

**Figure 2:**
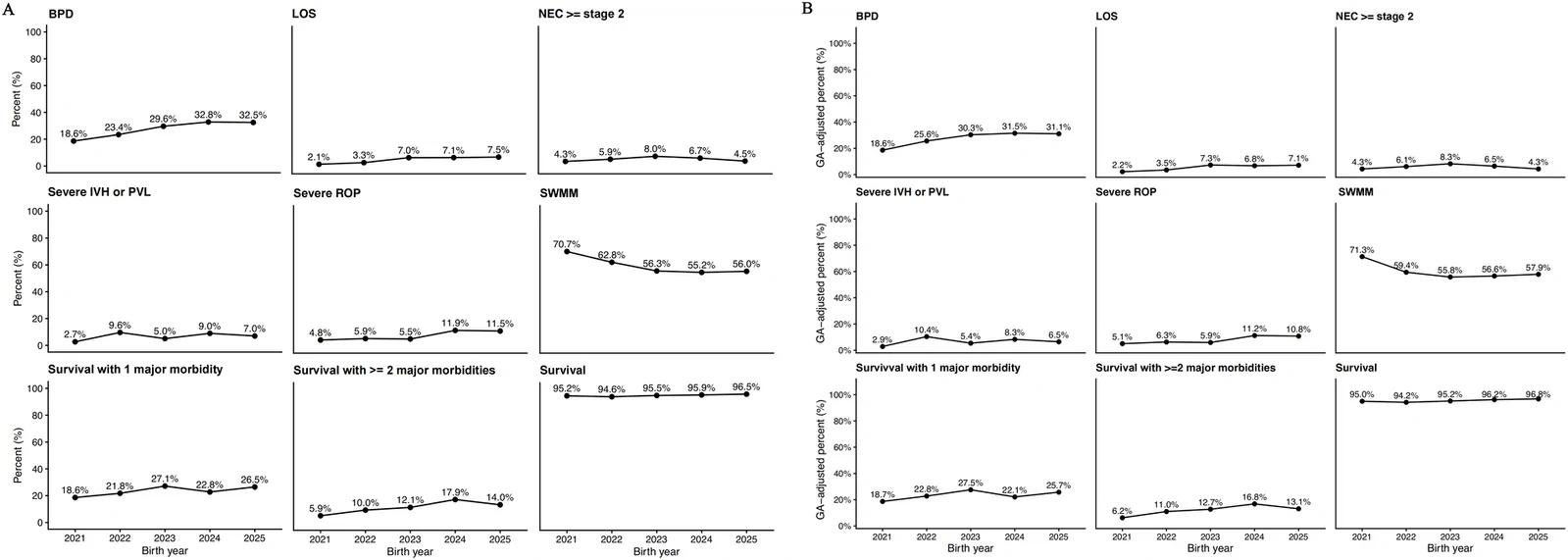
**(A)** Crude temporal trends and **(B)** gestational age-adjusted temporal trends in survival, major morbidities, and survival without major morbidity (SWMM) among very preterm infants receiving complete care from 2021 to 2025.

Figures 2A,B and Supplementary Table S4 illustrate crude and GA-adjusted temporal trends in the complete-care cohort from 2021 to 2025. Overall survival remained consistently high in both analyses. In crude analyses, BPD increased from 18.6% to 32.5%, severe ROP from 4.8% to 11.5%, and SWMM declined from 70.7% to 56.0%. After GA adjustment, the corresponding annual rates showed similar patterns. In GA-adjusted linear trend analyses, survival remained stable over time (OR per year, 1.16; 95% CI, 0.93–1.43). BPD increased over time (OR per year, 1.22; 95% CI, 1.09–1.36), as did severe ROP (OR per year, 1.30; 95% CI, 1.09–1.54). SWMM decreased over time (OR per year, 0.84; 95% CI, 0.75–0.93). LOS and survival with ≥2 major morbidities also showed significant increasing trends, whereas NEC ≥ stage II, severe brain injury, and survival with 1 major morbidity did not show statistically significant linear trends after GA adjustment.

### Factors associated with SWMM

**Figure 3:**
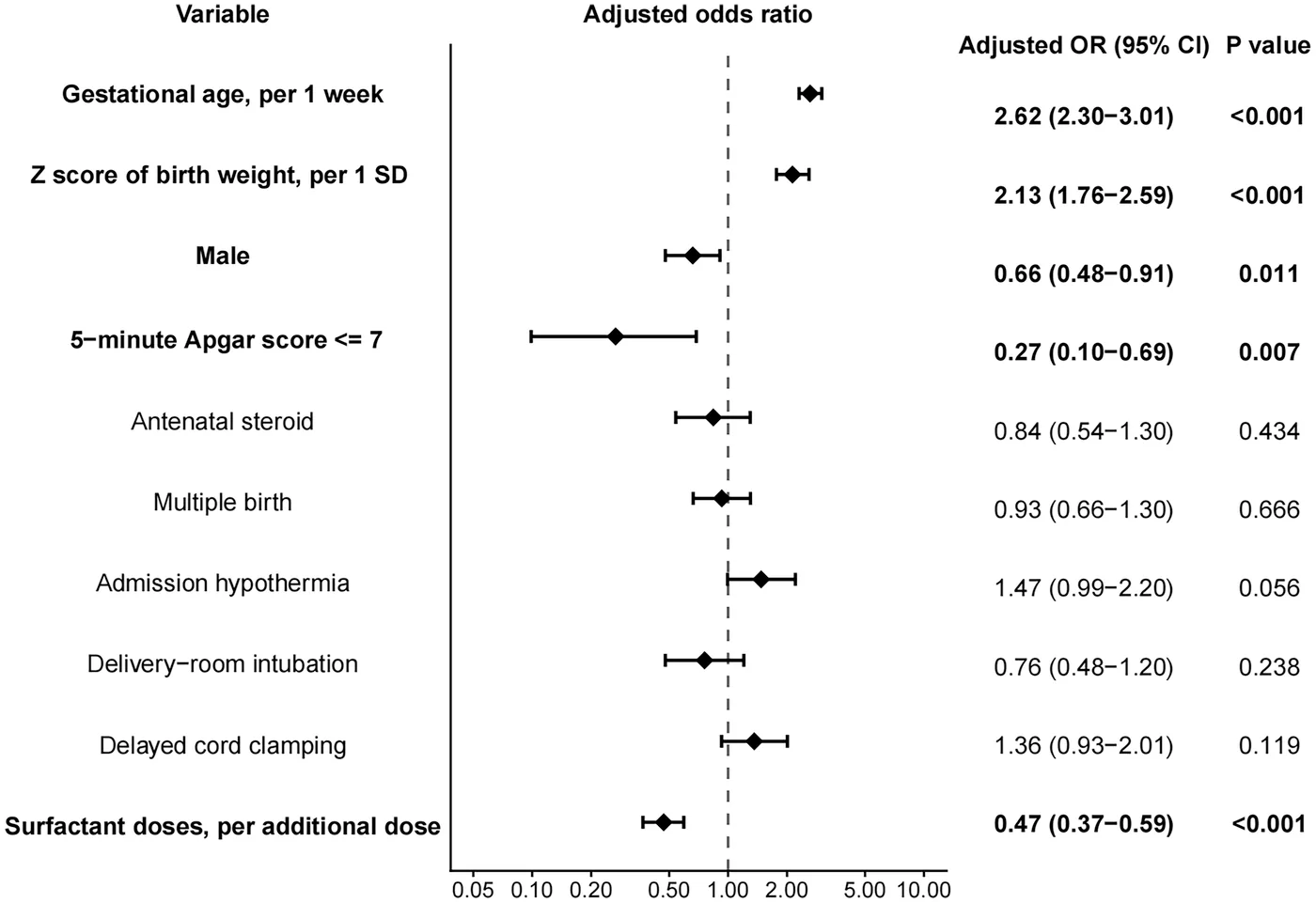
Factors associated with survival without major morbidity (SWMM) among very preterm infants receiving complete care.

Multivariable logistic regression analysis (Figure 3) showed that higher GA (per 1 week: OR 2.62, 95% CI 2.30–3.01) and greater BW z score (per 1 SD: OR 2.13, 95% CI 1.76–2.59) were associated with higher odds of SWMM. In contrast, male infant (OR 0.66, 95% CI 0.48–0.91), surfactant doses (per additional dose: OR 0.47, 95% CI 0.37–0.59) and 5-minute Apgar score ≤7 (OR 0.27, 95% CI 0.10–0.69) were associated with lower odds.

No evidence of substantial multicollinearity was observed in the primary model, with VIFs ranging from 1.02 to 1.26 (Supplementary Table S1). In sensitivity analyses, GA remained strongly associated with higher odds of SWMM after exclusion of BW z score (adjusted OR 2.29 per week, 95% CI 2.04–2.59), while BW z score remained independently associated with SWMM after exclusion of GA (adjusted OR 1.42 per SD, 95% CI 1.22–1.66; Supplementary Table S2). The associations of a 5-minute Apgar score ≤7 and increasing surfactant doses with lower odds of SWMM remained consistent in both sensitivity models. The primary model showed good discrimination, with an AUC of 0.87 (95% CI 0.85–0.89), and a Brier score of 0.14 (Supplementary Table S3). Its discrimination and overall information criteria were superior to those of the models excluding BW z score or GA.

### Survival outcomes stratified by care status and distribution of DAMA

Among all NICU-admitted VPIs, the overall survival rate was 90.8%, increasing to 95.5% among those receiving complete care. Survival improved markedly with increasing GA.

The proportion of DAMA was 6.7% overall and decreased substantially with increasing GA, from 30.0% at ≤25 weeks to 1.9% at 31 weeks. Among DAMA infants, the estimated post-DAMA survival rate based on prespecified criteria was low (24.4%) (Table 3).

**Table 3:** Survival outcomes and DAMA distribution among VPIs, stratified by GA.

Variables	Total	GA ≤25	GA 26	GA 27	GA 28	GA 29	GA 30	GA 31
Survival^a^^,^^d^, *n* (%)	19/78 (24.4)	0/12 (0.0)	0/6 (0.0)	3/14 (21.4)	5/17 (29.4)	6/10 (60.0)	3/12 (25.0)	2/7 (28.6)
Survival^b^, *n* (%)	1,064/1,172 (90.8)	20/40 (50.0)	33/48 (68.8)	84/98 (85.7)	158/180 (87.8)	195/202 (96.5)	218/234 (93.2)	356/370 (96.2)
Survival^c^, *n* (%)	1,045/1,094 (95.5)	20/28 (71.4)	33/42 (78.6)	81/84 (96.4)	153/163 (93.9)	189/192 (98.4)	215/222 (96.8)	354/363 (97.5)
DAMA^b^, *n* (%)	78/1,172 (6.7)	12/40 (30.0)	6/48 (12.5)	14/98 (14.3)	17/180 (9.4)	10/202 (5.0)	12/234 (5.1)	7/370 (1.9)

SWMM, survival without major morbidity; DAMA, discharge against medical advice; GA, gestational age; VPI, very preterm infant.

a Estimated among infants subject to DAMA (*n* = 78).

b Calculated among all NICU-admitted infants (*n* = 1,172).

c Calculated among infants receiving complete care (*n* = 1,094).

d Post-DAMA survival was estimated using prespecified criteria: infants requiring ventilatory support, inotropes, or exclusive parenteral nutrition at discharge were considered non-survivors.

## Discussion

This study systematically evaluated survival, major morbidities, SWMM, temporal trends, and factors associated with SWMM among VPIs admitted to the NICU of GDWCH from 2021 to 2025. Survival remained consistently high over the study period, whereas SWMM remained suboptimal and declined in parallel with increasing rates of BPD and severe ROP. Lower biological maturity, perinatal instability, and a greater early respiratory disease burden were independently associated with lower odds of achieving SWMM.

The survival rates in our cohort (95.5% in the complete-care cohort and 90.8% among all admissions) were comparable to those reported by recent national and international studies of VPIs. The 2022 Shenzhen multicenter data reported an overall VPI survival rate of 90.46% for all admissions (16). Similarly, the 2019 Chinese Neonatal Network (CHNN) data showed survival rates of 95.4% in the complete-care cohort and 87.6% among all admissions (15). In terms of international evidence, Dutch registry data collected from 2016 to 2021 revealed a VPI survival rate of 92.9% (26). These findings indicate that survival outcomes at our center are in line with those achieved in other contemporary tertiary NICUs (9–12, 16, 27–29).

Despite high survival, the SWMM rate of 59.9% in the complete-care cohort indicates a persistent gap. This finding underscores the importance of incorporating SWMM as a complementary quality indicator. The observed SWMM rate was comparable to those reported by the CHNN (57.2%) (15) and the Shenzhen cohort (65.79%) (16), but higher than the pooled estimate of 47% reported in a global meta-analysis (13). However, direct comparisons should be interpreted cautiously because of differences in GA distribution, patient case mix, definitions of major morbidities, DAMA ascertainment, and center-specific clinical practices (12–14).

To facilitate early risk stratification, the multivariable analysis was restricted to perinatal and early postnatal variables. Five factors—GA, birth weight z score, male sex, 5-minute Apgar score ≤7, and the number of surfactant doses—were independently associated with SWMM. These variables represent three closely related domains that influence neonatal outcomes: biological maturity and fetal growth, perinatal physiological stability, and the burden of early respiratory disease. Consistent with previous studies, lower GA and poorer fetal growth were associated with reduced odds of SWMM, underscoring the central role of developmental immaturity in adverse neonatal outcomes (12, 16, 17, 30, 31). Likewise, a low 5-minute Apgar score likely reflects compromised perinatal adaptation and greater physiological instability during the immediate postnatal period. Although repeated surfactant administration was also associated with lower odds of SWMM, the need for multiple surfactant doses most likely serves as a surrogate marker of greater pulmonary immaturity, more severe respiratory distress syndrome, and a higher early respiratory disease burden. These findings suggest that future efforts should focus on delivery-room stabilization and early respiratory management, particularly for infants born at ≤29 weeks’ gestation.

One clinically important finding of this study was the increasing burden of BPD and severe ROP despite persistently high and stable survival, which may also be the principal contributors to the observed decline in SWMM. Several factors may explain these trends. First, changes in case mix may have increased the risk for major morbidities. Although adjustment for GA attenuated differences in biological maturity across years, residual differences in illness severity and antenatal risk profile may not have been fully captured. Second, BPD and severe ROP share multiple established risk factors, including prolonged respiratory support, cumulative oxygen exposure, systemic inflammation, impaired postnatal growth, and transfusion exposure (32, 33). Because these morbidities arise from overlapping pathways, they may increase concurrently when exposure to these shared risk factors increases. Third, improvements in retinal imaging technology and increasing experience with standardized ROP screening may also have contributed to the temporal increase in severe ROP.

DAMA represents a critical variable in real-world outcome analyses of VPIs. In this study, DAMA was disproportionately prevalent among the lowest GA groups, and estimated post-DAMA survival was 24.4% based on prespecified criteria. This estimate should be interpreted cautiously because systematic post-discharge follow-up data were unavailable. Nevertheless, reporting DAMA separately is important because excluding DAMA infants may overestimate real-world outcomes among all NICU-admitted VPIs, whereas including DAMA infants without distinction may obscure outcomes among complete-care infants (23, 24). DAMA should be managed as part of high-risk neonatal care rather than solely an external socioeconomic issue. Early structured interventions within the first 24–72 h after birth may help reduce care withdrawal, including multidisciplinary meetings, repeated prognostic counseling, financial support, and psychosocial support.

This study has several strengths. It was based on a large, contemporary inborn cohort, minimizing potential confounding related to interhospital variations in care. By reporting survival, SWMM, major morbidities, and DAMA concurrently, the study provides a more comprehensive assessment of short-term neonatal outcomes than survival alone. Furthermore, the use of GA-adjusted temporal trend analyses, prespecified multivariable modeling, and complementary model diagnostics and sensitivity analyses strengthens the robustness and interpretability of the findings. Several limitations should also be acknowledged. First, although temporal trends were adjusted for GA, residual confounding cannot be excluded. Second, as this was a single-center study, the findings may not be fully generalizable to different NICUs. Third, SWMM reflects only short-term in-hospital outcomes and does not capture long-term outcomes. Finally, post-DAMA survival was estimated using prespecified clinical criteria rather than confirmed through systematic follow-up.

In conclusion, survival among inborn VPIs at our center remained consistently high throughout the study period, whereas SWMM remained suboptimal and declined in parallel with increasing rates of BPD and severe ROP. Gestational maturity, perinatal physiological stability, and the severity of early respiratory disease were the principal factors associated with SWMM, underscoring the importance of gestational age-stratified care, optimized perinatal stabilization, and lung-protective respiratory and oxygen management. Our findings also highlight the importance of considering DAMA when evaluating neonatal outcomes to provide a more accurate assessment of real-world clinical performance. Together, these findings support the use of SWMM as a complementary quality indicator alongside survival and may help inform quality-improvement initiatives, risk stratification, and family counseling for very preterm infants.

## Data Availability

De-identified data may be made available from the corresponding author upon reasonable request and with permission from the relevant institutional ethics committee.
