# Supplementary material for: Survival without major morbidity in very preterm infants (<32 weeks’ gestation): prevalence, temporal trends, and associated factors in a single-center retrospective cohort
Source: Front Pediatr. 2026 Aug 28;14:1871207. doi: 10.3389/fped.2026.1871207 (PMC13561976; doi:10.3389/fped.2026.1871207)
Supplement: Supplementary file 1 [file Table1.docx]

Supplementary

Table S1. Variance inflation factors for predictors included in the multivariable logistic regression model for SWMM

| Variable | VIF |
| --- | --- |
| GA | 1.24 |
| Male | 1.02 |
| BW z score | 1.26 |
| 5-minute Apgar score ≤7 | 1.14 |
| ANS | 1.02 |
| Multiple birth | 1.05 |
| Admission hypothermia | 1.14 |
| Delivery-room intubation | 1.20 |
| DCC | 1.05 |
| PS doses | 1.08 |

Note: Multicollinearity was assessed using VIFs, with VIF values <5 indicating no evidence of substantial multicollinearity.

Abbreviations: VIF, variance inflation factor; GA, gestational age; BW, birth weight; ANS, antenatal steroids; DCC, deferred umbilical cord clamping; PS, pulmpnary surfactant; SWMM, survival without major morbidity.

Table S2. Sensitivity analyses of the multivariable logistic regression model for SWMM among VPIs receiving complete care.

| Variable | Primary model, adjusted OR (95% CI); P value | Excluding BW z score, adjusted OR (95% CI); P value | Excluding GA, adjusted OR (95% CI); P value |
| --- | --- | --- | --- |
| GA, per 1 week | 2.62 (2.30–3.01); <0.001 | 2.29 (2.04–2.59); <0.001 | — |
| Male | 0.66 (0.48–0.91); 0.011 | 0.70 (0.52–0.96); 0.026 | 0.77 (0.59–1.01); 0.060 |
| BW z score, per 1 SD | 2.13 (1.76–2.59); <0.001 | — | 1.42 (1.22–1.66); <0.001 |
| 5-minute Apgar score ≤7 | 0.27 (0.10–0.69); 0.007 | 0.28 (0.11–0.70); 0.008 | 0.36 (0.16–0.77); 0.011 |
| ANS | 0.84 (0.54–1.30); 0.434 | 0.81 (0.53–1.24); 0.335 | 1.06 (0.74–1.51); 0.745 |
| Multiple birth | 0.93 (0.66–1.30); 0.666 | 0.89 (0.65–1.24); 0.497 | 1.18 (0.89–1.57); 0.246 |
| Admission hypothermia | 1.47 (0.99–2.20); 0.056 | 1.02 (0.70–1.48); 0.916 | 1.13 (0.81–1.58); 0.477 |
| Delivery-room intubation | 0.76 (0.48–1.20); 0.238 | 0.78 (0.50–1.22); 0.273 | 0.63 (0.43–0.92); 0.016 |
| DCC | 1.36 (0.93–2.01); 0.119 | 1.31 (0.90–1.91); 0.157 | 1.29 (0.93–1.82); 0.134 |
| PS doses, per additional dose | 0.47 (0.37–0.59); <0.001 | 0.50 (0.40–0.63); <0.001 | 0.33 (0.26–0.40); <0.001 |

Abbreviations: GA, gestational age; BW, birth weight; ANS, antenatal steroids; DCC, deferred umbilical cord clamping; PS, pulmpnary surfactant; SWMM,survival without major morbidity; VPI, very preterm infant.

Table S3. Performance of the multivariable logistic regression models for SWMM.

| Model | Complete-case, n | SWMM, n | AUC (95% CI) | Brier score | Hosmer-Lemeshow P value | AIC | BIC | Converged |
| --- | --- | --- | --- | --- | --- | --- | --- | --- |
| Primary model | 1094 | 655 | 0.87 (0.85–0.89) | 0.14 | 0.001 | 987.80 | 1042.77 | Yes |
| Excluding BW z score | 1094 | 655 | 0.85 (0.83–0.87) | 0.15 | 0.006 | 1052.01 | 1101.98 | Yes |
| Excluding GA | 1094 | 655 | 0.75 (0.72–0.78) | 0.20 | 0.036 | 1289.38 | 1339.36 | Yes |

Abbreviations: GA, gestational age; BW, birth weight; SWMM, survival without major morbidity; AUC, area under the receiver operating characteristic curve; AIC, Akaike information criterion; BIC, Bayesian information criterion.

Note: AUC reflects model discrimination, the Brier score overall prediction accuracy, the Hosmer-Lemeshow test together with the calibration intercept and slope model calibration, and AIC/BIC relative model fit accounting for model complexity. Higher AUC and lower Brier score, AIC, and BIC indicate better model performance, whereas a calibration intercept close to 0 and calibration slope close to 1 indicate better calibration.

Table S4. Crude and GA-adjusted temporal trends in from 2021 to 2025 among VPIs receiving complete care.

| Outcome | N | Events | Crude OR per year | 95% CI | Crude P for trend | GA-adjusted OR per year | 95% CI | GA-adjusted P for trend |
| --- | --- | --- | --- | --- | --- | --- | --- | --- |
| BPD | 1094 | 303 | 1.21 | 1.10-1.34 | <0.001 | 1.22 | 1.09-1.36 | <0.001 |
| LOS | 1094 | 60 | 1.34 | 1.10-1.64 | 0.004 | 1.31 | 1.07-1.61 | 0.009 |
| NEC ≥ stage II | 1094 | 65 | 1.03 | 0.85-1.23 | 0.790 | 1 | 0.84-1.21 | 0.970 |
| Severe brain injury | 1094 | 76 | 1.12 | 0.95-1.33 | 0.185 | 1.08 | 0.90-1.29 | 0.400 |
| Severe ROP | 1094 | 89 | 1.33 | 1.12-1.57 | <0.001 | 1.3 | 1.09-1.54 | 0.003 |
| SWMM | 1094 | 655 | 0.86 | 0.78-0.94 | <0.001 | 0.84 | 0.75-0.93 | 0.001 |
| Survival with 1 major morbidity | 1094 | 255 | 1.09 | 0.99-1.21 | 0.089 | 1.08 | 0.97-1.20 | 0.171 |
| Survival with ≥2 major morbidities | 1094 | 135 | 1.27 | 1.11-1.45 | <0.001 | 1.25 | 1.08-1.45 | 0.003 |
| Survival | 1094 | 1045 | 1.1 | 0.89-1.36 | 0.373 | 1.16 | 0.93-1.43 | 0.185 |

Note: Temporal trends were assessed using logistic regression models with birth year entered as a continuous variable. GA-adjusted models included GA as a continuous covariate. OR >1 indicates an increasing trend over time, whereas OR <1 indicates a decreasing trend.

Abbreviations: GA, gestational age; SWMM, survival without major morbidity; NEC, necrotizing enterocolitis; ROP, retinopathy of prematurity; BPD, bronchopulmonary dysplasia; LOS, late-onset sepsis; VPI, very preterm infant.
